# Inverters with Different Load Configurations and a Two-Input Multiplexer Based on IGZO NMOS TFTs

**DOI:** 10.3390/nano16020078

**Published:** 2026-01-06

**Authors:** Isai S. Hernandez-Luna, Jimena Quintero, Arturo Torres-Sanchez, Rodolfo García, Miguel Aleman, Norberto Hernandez-Como

**Affiliations:** 1 Centro de Nanociencias y Micro y Nanotecnologías, Instituto Politécnico Nacional, Ciudad de México 07738, Mexico; ishernandezl@ipn.mx (I.S.H.-L.); atorressa@ipn.mx (A.T.-S.); 2 Universidad Politécnica de Tapachula, Tapachula Chiapas 30830, Mexico; 213087@uptapachula.edu.mx; 3 Centro Universitario UAEM Ecatepec, Universidad Autónoma del Estado de México, Ecatepec de Morelos 55020, Mexico; rzgarcial@uaemex.mx; 4 Centro de Investigación en Computación, Instituto Politécnico Nacional, Ciudad de México 07738, Mexico; maleman@ipn.mx

**Keywords:** a-IGZO, thin-film transistors, inverters, load configurations, multiplexer

## Abstract

Amorphous indium-gallium-zinc-oxide (a-IGZO) thin-film transistors (TFTs) have emerged as promising candidates for next-generation large-area and low-power electronics due to their high mobility, low leakage current, and compatibility with low-temperature fabrication on flexible or transparent substrates. In this work, we report the fabrication of bottom-gate a-IGZO NMOS TFTs using HfO_2_ as high-k gate dielectric and Mo top contacts. The devices were electrically characterized through capacitance–voltage (C–V) and current–voltage (I–V) measurements, from which key parameters were extracted. Based on these transistors, we designed, fabricated, and characterized inverters employing four different load configurations: resistive, diode, depletion, and pseudo-CMOS. A comparative analysis was performed in terms of voltage transfer characteristics (VTCs), gain, and noise margins, highlighting that depletion-load inverters offer the highest gain and robust noise margins. Finally, a two-channel multiplexer was designed and fabricated. The multiplexer was characterized under both square and sinusoidal input signals up to 1 kHz, demonstrating correct channel selection and robust switching behavior. These results confirm the potential of a-IGZO TFT-based circuits as building blocks for low-power and high-reliability digital and mixed-signal electronics.

## 1. Introduction

In recent years, oxide-based thin-film transistors (TFTs) have emerged as an attractive alternative to polycrystalline silicon devices for large-area electronics applications [1,2,3]. Among them, amorphous indium-gallium-zinc oxide (a-IGZO) TFTs stand out due to their high electron mobility, low leakage current, and low-temperature fabrication on flexible or transparent substrates. These features have consolidated IGZO TFTs as a key platform for applications in high-resolution displays, sensors, and low-power electronic circuits [2,4].

One of the essential components in digital electronics is the inverter, whose efficiency directly depends on the electrical properties of the transistors that compose it. In this context, parameters such as carrier mobility, threshold voltage (V_TH_), on/off current ratio (I_ON_/I_OFF_), subthreshold slope (SS), and series resistance (R_ON_) are critical. These are strongly influenced by material quality, device geometry, and fabrication methods. In particular, the use of high-k dielectrics, such as hafnium oxide (HfO_2_), has been shown to significantly improve channel electrostatic control, reduce operating voltage, and optimize gate capacitance [5,6].

Furthermore, the design and testing of inverters with different load configurations are essential to evaluate the integration potential of IGZO TFTs in logic circuits. Among the most studied architectures are resistive-load inverters, diode-load inverters, depletion-load inverters, and pseudo-CMOS inverters [7,8,9,10]. Each configuration presents advantages and limitations in terms of gain, power consumption, area, and fabrication complexity. Comparative analysis of these topologies makes it possible to identify key design trade-offs, such as lowering operating voltage, improving energy efficiency, or ensuring stability under continuous operation. Although IGZO TFTs have been extensively studied in applications such as pixel drivers and sensors, their integration into digital circuits like inverters, logic gates, and multiplexers still poses challenges [3,11,12,13,14].

In this work, we present the fabrication and electrical characterization of IGZO NMOS TFTs configured with a bottom-gate structure, using HfO_2_ as the gate dielectric and Mo as the top contacts. Different channel geometries (width/length ratios) were explored to analyze their effect on key parameters, and based on these transistors, two applications were implemented and tested: inverters and a multiplexer. Inverters with different load configurations were fabricated, and their differences in terms of gain, noise margin, and voltage transfer characteristics were analyzed, showing how each topology affects the logic behavior of the circuit. While several studies have investigated individual inverter configurations, few have simultaneously compared multiple load types fabricated under identical process conditions on the same substrate and further extended the analysis to a fully functional multiplexer. Our results demonstrate high gain values (greater than 50 for depletion-load inverters) and stable operation of a two-input multiplexer up to 1 kHz at low supply voltages (1–3 V), confirming the strong integration potential of the fabricated IGZO TFTs for compact, low-power, and high-reliability digital circuits.

## 2. Experimental Part

The fabrication process of IGZO thin-film transistors was carried out using a staggered bottom-gate structure with top source and drain contacts. The substrate employed was Corning Eagle XG glass (Corning Incorporated, Singapore), square-shaped with a side length of 1 inch and a thickness of 0.7 mm.

The process started with standard RCA cleaning of the substrate. A chromium/gold (Cr/Au) bilayer was deposited by electron-beam evaporation to form the gate electrode and subsequently patterned by potassium iodide–based wet etching. Hafnium oxide (HfO_2_) was deposited as the gate dielectric by atomic layer deposition (ALD) using tetrakis (diethylamide)hafnium (TDMAH) and H_2_O as precursors, at a substrate temperature of 150 °C and a precursor temperature of 85 °C for 200 cycles. The dielectric layer and contact openings were defined by wet etching using a buffered hydrofluoric acid solution.

The a-IGZO active layer was deposited at room temperature by RF sputtering from a commercial (Kurt J. Lesker, Jefferson Hills, PA, USA) In_2_O_3_:Ga_2_O_3_:ZnO (1:1:1 mol%) target in a pure Ar atmosphere.

The semiconductor was patterned by photolithography and HCl-based wet etching, followed by a thermal annealing step in air at 150 °C for 1 h. Although deposition in a pure Ar atmosphere can result in a relatively high carrier concentration, as reported in [15], the film in this study underwent post-deposition annealing in air, which promoted oxygen incorporation and helped stabilize the electrical properties. This process enables well-defined transfer characteristics and an adjusted carrier concentration within the optimal range for TFT operation, consistent with previously reported behavior [16].

Molybdenum (Mo) was deposited by RF sputtering at room temperature (5 mTorr, 100 W) to form the source and drain contacts, which were patterned using a standard lift-off process. For device definition, five mask layouts were designed using CAD tools, and lithography was carried out by direct writing with a Heidelberg system. A positive photoresist was spin-coated, developed in a potassium hydroxide–based solution, and post-baked. The minimum design rule employed in the layouts was 5 µm, including overlap, line spacing, line width, and channel length. Film thicknesses were measured by profilometry, yielding 60 nm for Cr/Au, 30 nm for HfO_2_, 10 nm for IGZO, and 200 nm for Mo.

**Figure 1:**
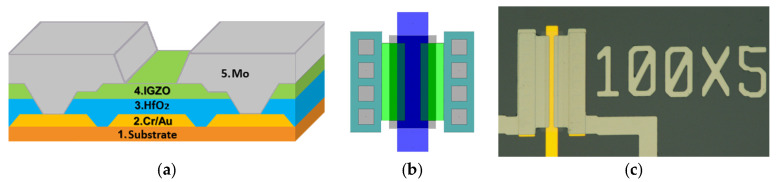
(**a**) Cross-sectional schematic of staggered bottom gate IGZO transistor; (**b**) Standard-cell layout design occupied in devices fabricated; and (**c**) top view image of IGZO TFT for W = 10 um and L = 5 um.

Finally, IGZO TFTs with different channel widths (W) and lengths (L) were fabricated, in Figure 1, show (a) the structure employed, (b) standard-cell layout design occupied in devices fabricated, and (c) a fabricated TFT with W = 100 µm and L = 5 µm. Additional metal–insulator–metal (MIS) structures were also included for material and interface evaluation. Electrical characterization of the devices was performed at room temperature using a Keithley 4200-SCS semiconductor parameter analyzer (Keithley Instruments, Solon, OH, USA).

## 3. Results and Discussion

**Figure 2:**
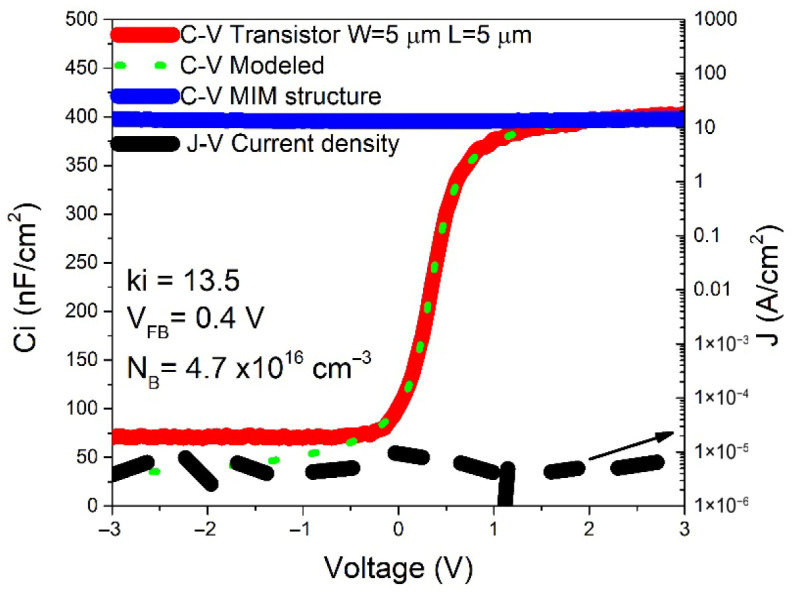
C–V curves of MIS and TFT structures, C–V curve modeled of TFT and current density of structures.

In Figure 2, the solid blue line shows the capacitance per unit area (Ci) as a function of voltage (C–V curve) for the MIM structure composed of Cr/Au/HfO_2_/Mo. The measurement was performed with a voltage sweep from −3 V to +3 V, at a frequency of 1 MHz and an AC signal amplitude of 50 mV, ensuring operation in the high-frequency regime. The dielectric constant (k_i_) was calculated using the parallel-plate capacitor model: Ci = ε0ki/xi; where xi = 30 nm is the thickness of the HfO_2_ dielectric and ε0 is the permittivity of free space in vacuum [6]. The extracted value, ki = 13.5, is consistent with reported values [6].

In Figure 2, the solid red line shows the C–V curve of a transistor with W = 5 µm and L = 5 µm. In this case, the gate terminal was biased while the drain and source were connected to the reference terminal of the measurement system. The transition between depletion and accumulation regions, similar to a MIS capacitor, is clearly observed. In the strong accumulation regime, the measured capacitance corresponds to the dielectric capacitance between the gate electrode and the accumulated charge in the semiconductor [17]. As expected, this value coincides with the capacitance measured in the MIS structure, confirming the dielectric constant of 13.5.

From the transistor C–V curve, the carrier concentration in the a-IGZO film was estimated using the 1/C^2^ slope method [18], yielding NB = 4.7 × 10^16^ cm^−3^. The green dashed line in the same figure corresponds to the modeled C–V curve, obtained by considering two capacitances in series: the dielectric capacitance and the space-charge capacitance of the semiconductor. The flat-band voltage (V_FB_) was determined from the flat-band capacitance (C_FB_) and its intercept on the voltage axis, giving V_FB_ = 0.4 V [18]. Since TFTs operate in accumulation mode regardless of channel material, V_FB_ is often used as a reference to predict the threshold voltage (V_TH_) in devices fabricated with the same technology [19].

Also in Figure 2, the black dashed line shows the current density measured across the MIS structure. No noticeable change is observed within the measurement range, which demonstrates the suitability of high-k dielectrics such as HfO_2_ for suppressing leakage currents, reducing the required operating voltages in AOS TFTs, and enhancing effective mobility [20,21].

**Figure 3:**
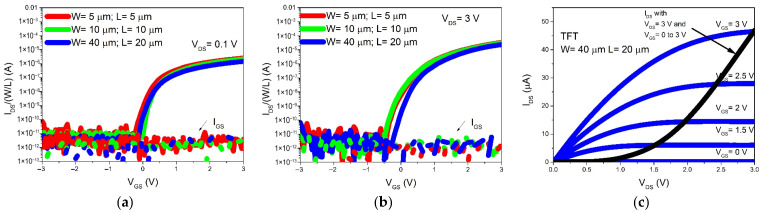
a-IGZO TFT transfer characteristic measured at (**a**) V_DS_ = 0.1 V, linear regime; (**b**) V_DS_ = 3 V, saturation regime; (**c**) Output characteristic for a width W = 40 um and length L = 20 um.

Figure 3 presents the logarithmic transfer characteristics of the transistors under (a) linear (V_DS_ = 0.1 V) and (b) saturation (V_DS_ = 3 V) regimes, for gate voltages swept from −3 V to +3 V. The on/off switching behavior is clearly observed, with similar slopes and shapes across devices of different channel dimensions. The measured gate leakage current (I_GS_) remains below 10 pA.

In Figure 3c, the output characteristics are shown (blue lines), obtained by sweeping V_DS_ up to 3 V while increasing V_GS_. The saturation transfer curve is overlaid, demonstrating a consistent fit between the two measurement modes.

Table 1 summarizes the extracted device parameters. From the saturation transfer curves, the threshold voltage (V_TH_) and field-effect mobility (µ_sat_) were obtained from the linear fit of I_DS_^1/2^ vs. V_GS_, based on the standard saturation equation [18]. Maximum current values are reported at V_GS_ = V_DS_ = 3 V. The on/off current ratio (I_ON_/I_OFF_) reaches ~10^7^ for all devices, while the subthreshold slope (SS), obtained from the inverse slope of log(I_DS_) vs. V_GS_ in the subthreshold region, remains below 260 mV/decade. The on-resistance R_ON_ was extracted from the output characteristics at V_GS_ = V_DS_ = 3 V. As expected, R_ON_ increases with larger channel dimensions, leading to reduced device performance.

To assess the reproducibility of the fabrication process, multiple transistors for each geometry were fabricated and characterized. The extracted electrical parameters showed limited device-to-device variation, with deviations below 5% in threshold voltage and 7% for mobility and other key parameters, confirming good process uniformity and yield across the sample.

Overall, the fabricated transistors exhibit a threshold voltage of ~0.7 V, average field-effect mobility of 25 cm^2^/V·s, high I_ON_/I_OFF_ ratios, and low SS values below 260 mV/decade. These results highlight the combined effect of the high-k dielectric, the optimized carrier concentration in the semiconductor, and the low series resistance on the overall device performance. It is important to emphasize that the electrical characteristics of TFTs arise from the interaction between all materials in the device stack and the specific sequence of fabrication step, both of which play a crucial role in achieving reproducible and stable transistor behavior.

Based on the results obtained from the electrical characterization of the fabricated transistors, we proceeded to design, fabricate, and characterize four typical inverter configurations and two-input multiplexer. These include the resistive-load inverter, the diode-load inverter, the depletion-load inverter, and the pseudo-CMOS inverter.

**Figure 4:**
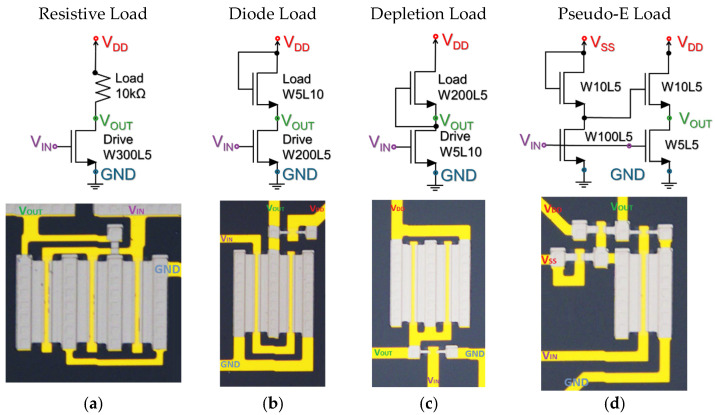
Schematics and fabricated devices of four inverter configurations: (**a**) resistive-load inverter, (**b**) diode-load inverter, (**c**) depletion-load inverter, and (**d**) pseudo-Enhancement Load.

Figure 4a shows the resistive-load inverter, where a commercial 10 kΩ external resistor is employed as the load element. Its main advantage lies in its simplicity of design and fabrication, since the inverter behavior depends only on a single driver transistor with W = 300 µm and L = 5 µm. However, this topology poses technological challenges for integration, as the fabrication process does not include dedicated materials for precisely defining resistive elements. Figure 4b illustrates the diode-load inverter. Here, the load is implemented using a transistor configured in diode mode W = 5 µm, L = 10 µm in series with the driver transistor W = 200 µm, L = 5 µm. This configuration eliminates the need for external resistors, simplifying integration, although it introduces a voltage drop across the diode-connected transistor, which limits the high-level output voltage (VOH).

Figure 4c shows the depletion-load inverter. In this case, the load is a wider channel transistor W = 200 µm, L = 5 µm operating in depletion mode, while the driver transistor is defined with W = 10 µm and L = 10 µm. Figure 4d presents the pseudo–Enhancement Load or pseudo-CMOS inverter, which uses two transistors in the load branch W = 10 µm, L = 5 µm each, combined with a driver transistor W = 100 µm, L = 5 µm and an additional input transistor W = 5 µm, L = 5 µm. This topology aims to emulate the behavior of a conventional CMOS inverter. Its main drawback is the higher design complexity and larger occupied area, since more transistors and interconnections are required.

**Figure 5:**
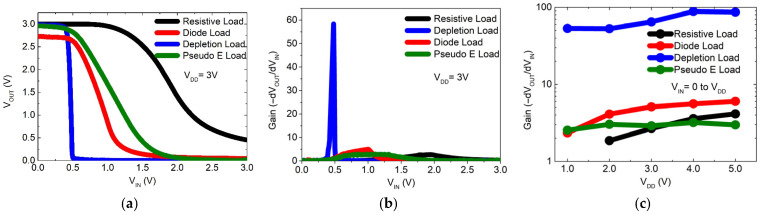
(**a**) Voltage transfer characteristics (VTCs) of inverters with different loads configuration at 3 V of supply voltage (VDD); (**b**) corresponding voltage gains at VDD = 3 V; (**c**) measured and calculated voltage gains of inverters versus VDD applied.

Figure 5a shows the voltage transfer characteristics (VTCs) of the fabricated inverters, while Figure 5b plots the voltage gain (−dV_OUT_/dV_IN_) with respect to the input voltage at V_DD_ = 3 V. The resistive-load inverter (black line) exhibits a wide transition with a moderate slope, indicating reduced noise margins and low gain. Although functional, this configuration is not ideal for digital logic applications requiring fast and robust switching. Furthermore, the fact that the curve does not reach 0 V (VOL) implies a high static power consumption and poor digital performance.

The diode-load inverter (red line) shows a steeper slope in the switching region compared to the resistive-load case. The transition is more abrupt, improving the noise margin and the definition of the switching point. However, significant static power consumption remains, and the high-level output voltage does not fully reach V_DD_ due to the diode drop. The pseudo-CMOS inverter (green line) exhibits a slope comparable to that of the diode-load inverter, showing similar gain and static consumption. While its digital behavior improves integration compared to the resistive design, it still does not reach the performance of a conventional CMOS inverter. Optimization of transistor dimensions is necessary to enhance its performance while reducing occupied area. The depletion-load inverter (blue line) shows the steepest slope and the most abrupt switching behavior among the four topologies, indicating the highest gain and improved digital behavior. This results in better noise immunity and nearly ideal output voltage levels (0 V and V_DD_). Although fabrication requires precise control of the depletion-mode transistor, this configuration demonstrates the best performance for complex logic applications.

Figure 5c shows the gain variation of the inverters as a function of the supply voltage VDD. For the resistive-load inverter, the gain remains low across the entire voltage range (≈2–4), increasing slightly with V_DD_ but never reaching robust digital logic levels. The diode-load inverter shows higher gain ≈3–7 as V_DD_ increases, but still insufficient for high noise margins. The pseudo-CMOS inverter maintains a nearly constant and low gain ≈2–3 across all voltages, which, while stable, limits its usefulness for logic circuits. In contrast, the depletion-load inverter clearly outperforms the others: its gain starts above 50 at V_DD_ = 1 V and scales up to nearly 100 at V_DD_ = 5 V, highlighting its capability to operate over a wide voltage range while maintaining robustness and abrupt switching.

Table 2 summarizes the noise margins of the inverters at V_DD_ = 3 V. The resistive-load inverter, while functional, shows low gain and reduced noise margins, making it unreliable for robust digital applications. The diode-load inverter provides a significant improvement in the high-level noise margin (NMH), though the low-level margin (NML) remains limited. The depletion-load inverter offers the best performance, with outstanding NMH values and very narrow undefined regions, resulting in abrupt and precise transitions.

The pseudo-CMOS inverter exhibits nearly ideal logic levels, balanced noise margins, and a slightly wider undefined region compared to the depletion-load design, making it a reliable choice for integrated logic despite not reaching the same abruptness.

**Figure 6:**
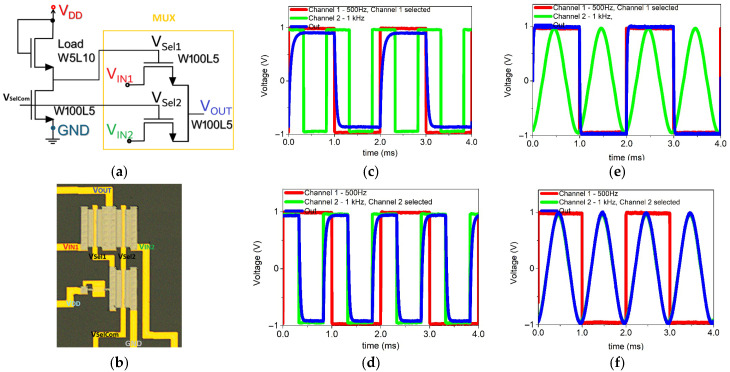
Two-input multiplexer implemented with IGZO TFTs: (**a**) circuit schematic and (**b**) optical micrograph of the fabricated device. Experimental characterization with input signals up to 1 kHz using square and sinusoidal waveforms: two square inputs at 500 Hz and 1 kHz with (**c**) channel 1 selected and (**d**) channel 2 selected; (**e**) square input at 500 Hz (channel 1) and sinusoidal input at 1 kHz (channel 2) with channel 1 selected; (**f**) same inputs with channel 2 selected.

Multiplexers (MUXs) are among the fundamental building blocks in digital system design, as they enable efficient signal routing and channel selection within more complex architectures such as arithmetic logic units (ALUs), memory addressing circuits, and programmable logic. Their integration is therefore essential for scaling from basic logic gates to functional digital systems. To explore the feasibility of implementing such blocks with oxide TFT technology, a two-input multiplexer was designed and fabricated using IGZO thin-film transistors. Figure 6a illustrates the proposed architecture, where the MUX is connected to a saturated-load inverter that generates the control signals (V_Sel1_ and V_Sel2_). Instead of requiring two independent selection inputs, the circuit employs a single common control terminal (V_SelCom_). V_SelCom_ signal is applied to the inverter, which automatically provides the complementary output needed to alternately activate V_Sel1_ or V_Sel2_. When V_SelCom_ is at a high logic level, the inverter produces a low level at V_Sel1_ and a high level at V_Sel2_, enabling the transistor associated with input V_IN2_ and transferring its signal to the output V_OUT_. Conversely, when V_SelCom_ is low, the inverter generates a high level at V_Sel1_ and a low level at V_Sel2_, enabling the transistor linked to V_IN1_. This strategy reduces the number of control terminals to a single selection input, simplifying both the design and the operation of the circuit. In each case, the output V_OUT_ corresponds to the signal applied at the selected input (V_IN1_ or V_IN2_). The transistors employed in the MUX have dimensions of W = 100 µm and L = 5 µm, ensuring low on-resistance and efficient signal transfer. Figure 6b presents the fabricated MUX together with the inverter that provides the selection signal.

The experimental characterization of a two-input multiplexer implemented with oxide thin-film transistors (TFTs) was carried out, evaluating its performance with test signals up to 1 kHz and using different waveforms, including both square and sinusoidal inputs, the plots show the input signals channel 1/V_IN1_ and channel 2/V_IN2_ along with the multiplexer output. In Figure 6c,d, the operation of the multiplexer is demonstrated for square-wave inputs at 500 Hz and 1 kHz. The output signal (blue trace) accurately reproduces the corresponding input waveform, confirming that the circuit remains stable even at higher input frequencies. In Figure 6e,f, the multiplexer is tested with mixed input signals: a 500 Hz square wave on channel 1 and a 1 kHz sinusoidal wave on channel 2. When the selector activates channel 1, the output follows the square waveform; conversely, when channel 2 is selected, the output tracks the sinusoidal waveform.

It is worth noting that the saturated-load inverter used to generate the selection signals was biased with a supply voltage of 1.5 V, which ensured well-defined high and low states for the control channels (VSel1 and VSel2). In addition, the input signals applied to V_IN1_ and V_IN2_ were limited to a maximum level of 1 V, making the circuit compatible with current low-voltage electronic applications and demonstrates the capability of circuits to handle mixed-signal operation, processing both digital and analog inputs.

**Figure 7:**
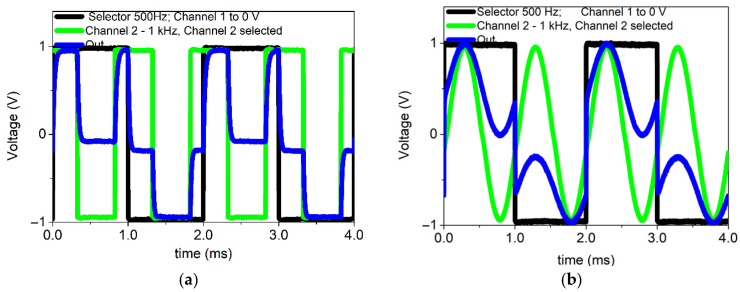
Shows the operation of the multiplexer when channel 2 is selected using a 500 Hz square-wave control signal (black trace). The input applied to channel 2 is either (**a**) a 1 kHz square wave or (**b**) a 1 kHz sinusoidal wave (green trace), and the resulting output signal is shown in blue line.

Figure 7a shows the operation of the multiplexer when channel 2 with a 1 kHz square wave input (green trace) is activated by the selector with a 500 Hz square wave (black trace) resulting in the corresponding output signal (blue trace). The observed behavior confirms the correct switching functionality of the device: when the selector is at a high logic level, the output faithfully follows the input signal, while at a low logic level on the selector, the output switches to the other predefined state. The output exhibits well-defined logic levels of ≈1 V (logic “1”) and ≈0 V (logic “0”), although transient changes to negative values ≈−1 V are also observed. These changes do not represent valid logic states but are attributed to undershoot effects during channel selection. Nevertheless, from a logic perspective, the device continues to operate correctly as a multiplexer. Such effects are characteristic of TFT-based circuits and depend on both the circuit topology and load conditions.

Finally, Figure 7b shows the case where a sinusoidal input is applied to channel 2. During intervals when the selector is at a high logic level, the output reproduces the sinusoidal input, confirming correct channel switching. In contrast, when the selector is at a low logic level, the output is decoupled from the sinusoidal input, validating the selection functionality of the device. The output preserves the general shape of the sinusoidal waveform but with reduced amplitude and slight distortions. These deviations can be attributed to resistive losses, parasitic capacitances, and intrinsic gain limitations of oxide TFTs. 

Undershoot effects during switching are also present. Overall, these results demonstrate that the multiplexer is not only capable of handling digital signals but can also process low-frequency analog signals, extending its potential application to mixed-signal systems in flexible and low-power electronics platforms.

Although the operational frequency of the fabricated multiplexer is limited to approximately 1 kHz, this restriction primarily arises from parasitic capacitances at the IGZO TFT interconnections and the relatively high output resistance of the diode-load driver stage. Higher frequencies could be achieved through improved device scaling, reduced channel length, or the adoption of self-aligned structures to minimize overlap capacitance. Nonetheless, the achieved operation frequency is consistent with previously reported oxide-TFT multiplexers [20], validating the potential of these devices for low-frequency signal processing and control in flexible or sensor-oriented electronics. The relevance of TFT-based multiplexers is not limited to their role in proof-of-concept circuits but has also been demonstrated in advanced applications. As in the case of hybrid memory architectures, serial access schemes supported by multiplexers are essential for efficiently addressing and organizing large device arrays, as reported in Memristor-CMOS systems [21]. These examples highlight the importance of exploring and demonstrating multiplexers using TFT technology, since they constitute indispensable building blocks for the scalability of circuits toward mixed applications in logic, memory, and sensing.

## 4. Conclusions

Based on the electrical characterization of the fabricated a-IGZO thin-film transistors, their performance was further evaluated in two main circuit structures: inverters with different load configurations and a two-input multiplexer. Among the inverter topologies investigated, the depletion-load configuration emerges as the most effective for demanding digital applications, providing the steepest voltage transfer characteristics, the highest gain values, and strong robustness in the high-level noise margin. Furthermore, the experimental characterization of the two-input multiplexer confirms correct channel selection and stable operation up to 1 kHz under both square and sinusoidal inputs. This demonstrates its capability to handle not only digital signals but also mixed-signal processing, thereby broadening its potential applications in flexible and low-power electronics. Overall, these findings validate the feasibility of a-IGZO TFT-based devices as practical building blocks for digital and mixed-signal circuits. Beyond serving as proof-of-concept prototypes, the depletion-load inverter and the multiplexer highlight the competitiveness of oxide TFT technology for scalable integration and future circuit design.

## Figures and Tables

**Table 1:** Summary of the parameters extracted from the TFTs.

W; L µm; µm	V_TH_ (V) (±5%)	µ_sat_ (cm^2^/Vs) (±7%)	I_Dmax_ (µA) (±7%)	I_ON_/I_OFF_	SS (mV/Decade) (±7%)	R_ON_ (±7%)
W = 5; L = 5	0.5	26.7	33.5	1.2 × 10^7^	260	37.5 kohm
W = 10; L = 10	0.7	25.2	28.4	1.0 × 10^7^	200	39.3 kohm
W = 40; L = 20	0.7	22.3	46.7	1.04 × 10^7^	222	62 kohm

**Table 2:** Summary of the parameters extracted from the inverter with different loads.

Inverter	Gain at 3 V	VOH	VIH	NMH	VIL	VOL	NML	Indeterminate Region
Resistive Load	2.6	2.99	2.36	0.63	1.33	0.45	0.88	1.03
Diode Load	5.1	2.73	1.22	1.51	0.49	0.04	0.45	0.73
Depletion Load	58.3	2.99	0.51	2.48	0.38	0.00	0.38	0.13
Pseudo-E Load	2.9	2.95	1.63	1.32	0.51	0.01	0.50	1.12

## Data Availability

The original contributions presented in this study are included in the article. Further inquiries can be directed to the corresponding author.
